# Pertussis: A Review of Disease Epidemiology Worldwide and in Italy

**DOI:** 10.3390/ijerph9124626

**Published:** 2012-12-11

**Authors:** Giovanni Gabutti, Maria Cristina Rota

**Affiliations:** 1 LHU 4 “Chiavarese”, Hygiene and Public Health O.U., Corso Dante, Chiavari (Ge) 163-16043, Italy; 2 National Center for Epidemiology, Surveillance and Health Promotion (CNEPS), National Institute of Health (ISS), Rome I-00161, Italy; E-Mail: rota@iss.it

**Keywords:** pertussis, epidemiology, disease, review

## Abstract

Pertussis continues to be a relevant public-health issue. The high coverage rates achieved have decreased the spread of the pathogen, but the waning of immunity implies a relevant role of adolescents and adults in the infective dynamics as they may represent a significant source of infection for unvaccinated or incompletely immunized newborns. The passive surveillance system is affected by many limitations. The underestimation of pertussis in adolescents, young adults and adults is mainly related to the atypical clinical characteristics of cases and the lack of lab confirmation. The real epidemiological impact of pertussis is not always perceived, anyway, the unavailability of comprehensive data should not hamper the adoption of active prophylactic interventions aimed at preventing the impact of waning immunity on pertussis. To avoid an increase of the mean age of acquisition of the infection, a booster dose of low-antigen content combined vaccine should be adopted in adolescents and adults. A decreased risk of infection in newborns can be achieved with the cocoon strategy, although the debate on this aspect is still open and enhanced surveillance and further studies are needed to fine-tune the pertussis prevention strategy.

## 1. Introduction

Pertussis is a human respiratory disease sustained by *Bordetella pertussis* and transmitted through Flügges droplets. The disease is highly infective and its basic reproduction number (Ro), which represents the number of secondary cases caused by each primary case in a population of fully susceptible subjects, is estimated to be particularly high [1,2,3].

The disease affects all age groups, particularly children, and is one of the most relevant causes of death in infants younger than 1 year of age. The incubation period usually lasts for 7–10 days (range 1–3 weeks) and the clinical features are related to the age of acquisition of infection, to any available immune level, and to antibiotic therapy. Besides, the severity of the disease is inversely related to the age of the patient; in unvaccinated children, pertussis has a typical course and can imply severe symptoms and complications. The prognosis can be particularly severe during the first and second year of life, when incidence, as well as hospitalizations and deaths are particularly high (case fatality rate: 0.2% and 4% in developed and developing countries, respectively) [4]. 

In immunized children, adolescents and adults, the disease may have a mild and aspecific course [5]; for this reason, in these subjects the disease is usually not diagnosed [6]. These subjects may represent a relevant source of infection for children, particularly for infants during their first year of life, when they are not yet completely immunized [7]. Several seroepidemiological surveys suggest a high incidence of the disease in adolescents and adults. 

The spreading of the infection can be stopped only by achieving high immunization coverage in the population (>92%). Immunity against pertussis, both natural and acquired by immunization, does not persist life-long; nowadays, immune protection is believed to wane after 4–12 years. Accordingly to these observations, epidemic outbreaks, have been registered in highly immunized populations, mainly in adolescents and adults [8,9]. Taking into account all the previous points, the purpose of this study was to assess and review the epidemiology of pertussis worldwide and in Italy.

## 2. Worldwide Epidemiology

Pertussis is a worldwide endemic-epidemic infectious disease, with outbreaks every 3–5 years and a summer-autumn seasonality. In the pre-antibiotic and pre-immunization era, both incidence and case fatality ratio were high; the disease mainly affected children younger than 5 years of age. The adoption of both antibiotic therapy and immunization has significantly decreased the number of cases as well as mortality [10]. 

Immunization against pertussis (combined with those against tetanus and diphtheria) has been included in the WHO’s Expanded Programme on Immunization (EPI) in 1974. Accordingly to data provided for 2008, the estimated worldwide rate of newborns immunized with three doses was about 82%. Nevertheless, WHO estimates that during 2008 about 16 million cases, 95% of these in developing countries, and about 195,000 deaths have occurred. In the same year, immunization has allowed avoidance of about 680,000 deaths [11]. 

**Figure 1:**
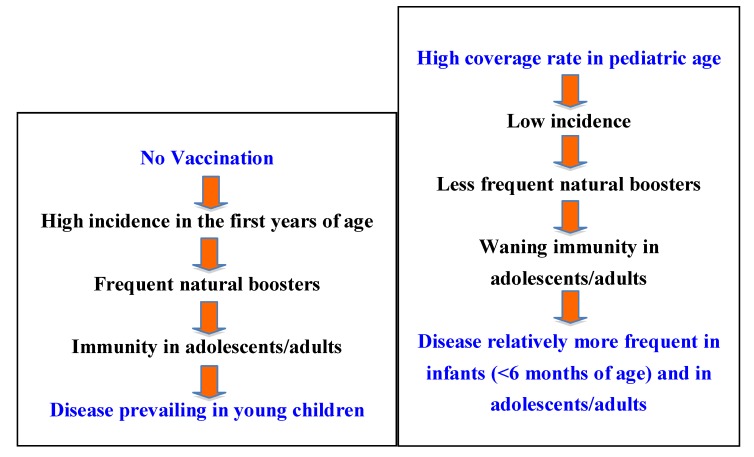
Pertussis: pre and post-immunization epidemiology related to the immunological pressure exerted by vaccination.

Whereas in developing countries data on the duration of immune protection are limited, several studies performed in developed countries have demonstrated that protection wanes after about 4–12 years [12,13,14]. As reported by Witt *et al*. in a recently published paper [15], their data suggest that the current schedule of acellular vaccine doses is insufficient to prevent outbreaks of pertussis and a markedly increased rate of disease from ages 8–12 years, proportionate to the interval since the last scheduled vaccine, was noted. Furthermore, as reported by Cherry [13], of particular concern at present is the fact that DTaP vaccines are less potent than DTP vaccines. Five studies conducted in the 1990s showed that DTP vaccines have greater efficacy than DTaP vaccines. During the last years an increasing number of cases has been registered in older children, adolescents and adults (Figure 1). Together with low immunity causing increased incidence of pertussis in several countries there are also some other explanations for this increasing trend, such as raised awareness; increased use of PCR; use of DTaP vaccines, less potent that DTP vaccines; and possible genetic changes in circulating strains of *Bordetella pertussis*, as reported by Cherry [13].

In USA, pertussis is a notifiable disease; data collected in the period 1990–2010, showed that the incidence (per 100,000 inhabitants) had a peak in 2004 and that there has been an increasing trend since 2007, which has now surpassed peak rates observed during 2004–2005. In the period 1990–2010, infants younger than 6 months of age have been heavily involved and in 2008–2009 the incidence in this age group had a 60% increase. In 2009, adolescents and adults accounted for approximately 40% of notified cases and, during the last years, children aged 7–10 years have sustained a growing proportion of cases (9%, 13%, 23.5% and 23% in 2006, 2007, 2008 and 2009, respectively) [16]. One of the latest outbreak has been registered in California where, in 2010, 9,477 cases occurred, corresponding to an incidence equal to 24.2/100,000 inhabitants; incidence was highest in infants aged <3 months. The 63% of hospitalizations involved subjects younger than 6 months of age. As a whole, 10 deaths occurred, nine in unimmunized infants (<2 months of age) and one in a subject (2 months of age) who had received one dose 15 days prior the onset of the disease [17,18].

More recently, a pertussis epidemic has been reported in the state of Washington. In the period Jan-16 Jun 2012, a total of 2,520 cases have been reported; the highest incidence was observed in infants aged <1 year and in children aged 10, 13 and 14 years. The rate of hospitalized infants aged <1 year has been equal to 21.9%; 41.2% of hospitalized infants were aged <2 months [19]. In USA, as well as in Canada, France, Germany, Brazil and Australia, household members, primarily parents, were the source of infection for infants [11,20,21,22]. 

Concerning Europe, reports from the European Centre for Disease Prevention and Control (ECDC) and from the Surveillance Network for vaccine preventable diseases (EUVAC-NET) have shown that, during 2003–2007, 43,482 cases have been notified (overall incidence: 4.1/100,000 inhabitants). High incidence rates have been registered in Norway, Sweden and Finland. The highest incidence rates have been reported in infants (35/100,000). In the age classes 1–4, 5–9, 10–14, 15–9 and >20 years, the 7%, 18%, 23%, 11% and 31% of cases have been notified, respectively. The majority of pertussis’ cases in infants <1 year old involved unvaccinated subjects, while the majority of cases notified in those aged 5–9 and 10–14 years were immunized with at least two doses. In the period 2003–2007, there were 2,777 hospitalizations (82/1,000 cases of pertussis) and 30 deaths (0.8/1,000 cases); the largest proportion of deaths (87%) was registered in infants [23]. Noteworthy, the accuracy of these data should be taken into account with caution as some countries reported only laboratory-confirmed cases whereas others reported clinical cases without laboratory confirmation.

Pertussis epidemiology has also been examined in selected Central and Eastern European countries and Turkey from 1945 to 2005. In the pre-vaccine era, pertussis incidence was high (180–651/100,000) with most cases occurring in pre-school children. During 1995–2005, when immunization coverage rate was high (80–98%), incidence sharply decreased (<3/100,000). An increase in age-specific incidence rates was registered in 5–14 year old children in some countries (e.g., Poland, Estonia, the Czech Republic) while incidence rates in <1 year of age subjects remained unchanged. In Central and Eastern European countries, despite high immunization coverage, pertussis infection persists and, in comparison to pre-vaccine era, the age distribution has shown a shift towards older children [24].

**Figure 2:**
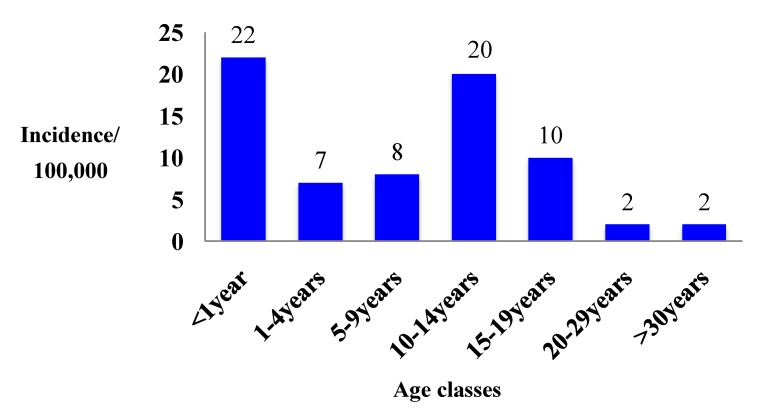
Incidence/100,000 cases of pertussis in Europe, stratified by age, 2009 (modified from EUVAC.NET annual report 2009)

In 2009, 20,591 cases of pertussis have been notified in Europe (4.9/100,000 inhabitants); the highest incidence rates were observed in Norway, Estonia, The Netherlands and Poland. Data were distributed between age groups as follows: 6%, 7%, 10%, 25%, 15%, 4%, 3% and 31% in subjects aged <1 year, 1–4, 5–9, 10–14, 15–19, 20–24, 25–29 and >30 years, respectively. Incidence was highest among infants (22/100,000) and among children aged 10–14 years. (20/100,000) (Figure 2). 

A proportion of 17%, 2% and 65% of cases was reported in unimmunized subjects, in subjects vaccinated with only one dose and in subjects vaccinated with at least two doses, respectively. The number of doses was unknown in 15% of cases.

Among unvaccinated subjects, 26% were newborns and 44% were adults > 20 years. The overall rate of hospitalization was equal to 104/1,000 cases of pertussis; the 29% and the 22% of hospital admissions involved newborns and subjects aged 10–14 years, respectively. During 2009, four deaths were notified, two in Bulgaria and two in UK; the latter involved unvaccinated newborns aged 6 and 7 months, respectively [25].

All these data clearly show that the burden of pertussis in terms of both incidence and hospitalizations primarily involves newborns. A research performed in the Netherlands in 2007, confirmed that older siblings (9–13 years old) and the mother play a relevant role in the transmission dynamics of the infection in newborns [20].

Seroepidemiological surveys have demonstrated that, also in Europe the infection is spreading in age classes considered as not usually involved in the pertussis’ spreading dynamics. The European Seroepidemiology Network (ESEN) has firstly standardized the lab methods allowing the comparison between surveys conducted in different countries [26]. Subsequently, high titres of antibody (>125 EU) to pertussis toxin (PT) have been shown to be a sensitive and specific indicator of recent infection in all age classes [27]. Recently, a serological study performed in five European countries (Finland, Germany, Italy, the Netherlands, UK) has estimated high incidence rates of infection among adolescents, young adults and even in older age classes [3].

## 3. Epidemiology in Italy

Since 1962, vaccination against pertussis with cellular vaccine has been recommended by the Italian Ministry of Health; however, for many years the coverage rate remained low, particularly in South Italy. Until 1991 the coverage rate did not exceed 40% and consequently was insufficient to control the spread of the infection [28].

The vaccination schedule currently adopted in Italy consists of three doses of acellular vaccines administered at 3, 5 and 11–12 months of age. From 1994 onwards, when acellular vaccines, considerably less reactogenic than cellular ones, were made available and included in combined products vaccination coverage has significantly improved. The coverage rate with three doses in children aged 12–24 months was equal to 88% in 1998, increased to 95% in 2003, reaching 96.6% in 2008. In the same year, the coverage rate was equal to 45.6%, 26.7% and 14.1% with three, four and five doses, respectively [29] among adolescents in their 16th year of age.

In Italy, pertussis is a mandatory notifiable disease [30]; the case definition is based on clinical diagnosis (a cough illness lasting more than 14 days with one of the following: paroxysm of coughing, inspiratory whoop or post-tussive vomiting without other apparent cause) and laboratory confirmation is not routinely looked for. Consequently, the detection of a classical pertussis disease in non-immunized infants does not give diagnostic problems, but several cases of atypical adult pertussis may be misdiagnosed. Therefore, the use of laboratory techniques, such as PCR/culture and serology, together with clinical symptoms of pertussis is desirable and could make the national incidence figures more valid.

**Figure 3:**
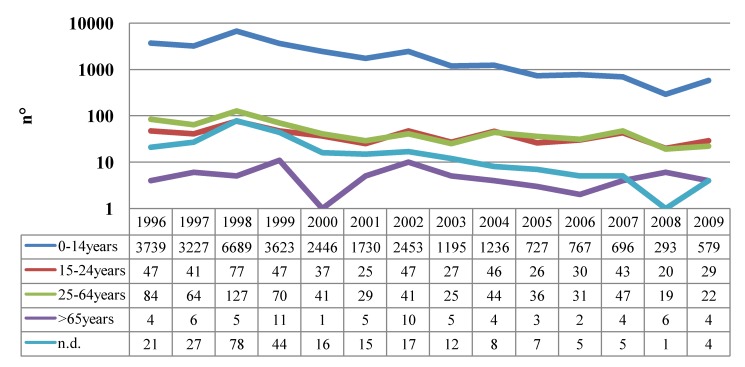
Pertussis: trend of notifications in Italy, 1996–2009 (source Ministry of Health).

The epidemiological trend has changed accordingly to the coverage rates achieved in different periods. The incidence rate fell from 38.4/100,000 inhabitants in the period 1956–1959 to 12.4/100,000 in the period 1971–1974, as a consequence of the adoption of the whole-cell vaccine in 1962 [28]. From the mid-1970s to the end of the 1980s, incidence rate increased (27.2/100,000 in the period 1986–1989). A peak in incidence was registered in 1987 (54.2/100,000) and in the subsequent years a constant decrease of cases was observed. Since 1994, the availability and the adoption of new acellular vaccines allowed to achieve and maintain high coverage rates and consequently lower incidence rates. A relevant decrease of complications and mortality in the first year of life and in the pre-school age has been achieved as well. In 1998, almost 7,000 cases of pertussis were notified; in 2005, notifications were 802 and during the following years this number decreased further [31] (Figure 3).

The impact of improved immunization strategy is well evident comparing different periods with different coverage rates: 1971–1989 (low vaccination coverage VC%), 1990–1996 (intermediate VC%) and 1998–2000 (high VC%). The achievement of high coverage has significantly decreased the incidence of pertussis in children aged less than 10 years. The comparison between different periods (1971–1989: low VC% vs 1998–2000: high VC%) shows a significant change in the percentage of cases in the different age classes. The proportional age distribution almost halved in the 0–4 year age class, has increased 1.5 times in the age class 5–9 year age class and has increased three-times in the 10–14 year age group [32].

The epidemiology of pertussis is changing in Italy as well as in other countries with a high coverage rate [33,34]. As a matter of fact, the immunological pressure exerted in the paediatric age has allowed a reduction not only in the incidence in children, but also in the chances of natural boosting. For these reasons, the disease is now increasing in adolescents or adults who have lost their immunological protection and in infants that have not yet begun or completed their primary immunization course [35]. A recent multicentre study performed in different geographical areas in Italy has evaluated both the humoral and the cell-mediated immunity against pertussis. This study suggests that *B. pertussis* continues to circulate in age groups that have been previously considered to be not involved in the circulation of this pathogen, particularly adolescents and adults [36].

Other epidemiological data show a progressive decrease of both mortality and lethality due to pertussis. In detail, in 1955 the number of deaths was 234, while the mortality fell down to 0.3, 0.1 and 0.02 /1,000,000 inhabitants in the 1970s, 1980s and 1990s, respectively. In the period 1990–2001, the overall number of deaths was 19; 14 of these were registered in children aged <12 months, three in 1 year old babies and two in children aged >2 years. The case fatality rate, equal to 100/10,000 cases of pertussis in 1955, decreased to 5 and 2/10,000 cases in the 1980s and the 1990s, respectively.

**Figure 4:**
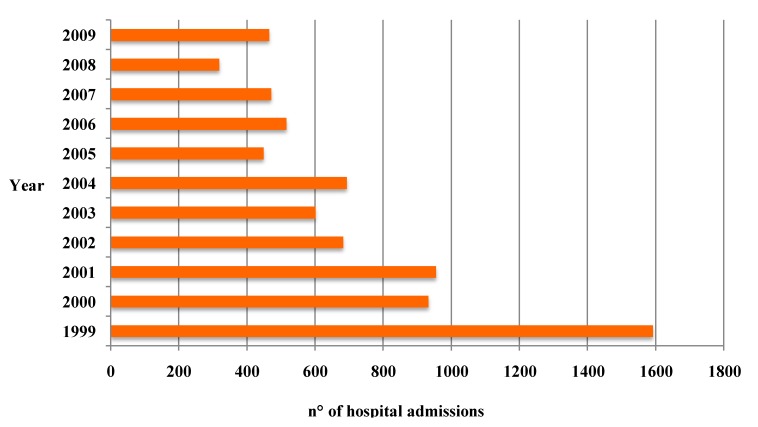
Pertussis: number of yearly hospitalizations in Italy, 1999–2009 (source Ministry of Health).

The national hospital discharge database is an additional source of data, useful to assess the impact of pertussis [37]. This database is available on the website of the Ministry of Health for the period 1999–2005; for the following years and until 2009, each annual report can be consulted. Until 2000, data were stratified as follows: <1, 1–4, 5–9, 10–14, 15–24, 25–44, 45–64, 65–75 and >75 years of life. Since 2001, hospitalizations in babies aged <1 year were further stratified in the groups: <1, 1–6 and 6–12 months. In the period 1999–2009, 7,768 hospitalizations due to pertussis have been registered (6,971 hospitalizations and 797 day hospital admissions) (Figure 4). Noteworthy, almost 64% of cases has been registered as pertussis due to unspecified pathogens (ICD9-CM code 033.9) and the 57.4% of hospital admissions involved subjects younger than 1 year of age. The mean length stay of hospitalizations was 5.6 days. 

## 4. Conclusions

Both national and international data show that pertussis continues to be a relevant public-health issue. The high coverage rates achieved in industrialized countries has certainly decreased the spreading of the pathogen; however, the waning of immunity implies a relevant role of adolescents and adults in the infective dynamics. Adolescents and adults may represent a significant source of infection for unvaccinated or incompletely immunized newborns [38,39].

The passive surveillance system, based on notification, is affected by limitations such as under or delayed reporting and under-diagnosis. Besides, the underestimation of pertussis in adolescents, young adults and adults is related to the atypical clinical characteristics of cases and the lack of lab confirmation. The clinical definition of *B. pertussis*’ infection is difficult due to the wide variability in disease expression; lab confirmation of pertussis should be standardized, single-serum serology in particular [40]. There is no general consensus on the lab assays to be used; serological tests, culture, molecular biology methods have both pros and cons [41].

The real epidemiological impact of pertussis is not always perceived; data on hospitalizations are not usually known, many deaths related to pertussis are not identified (particularly in newborns) and data on the elderly are scarce. The differences between passive and active surveillance systems have been demonstrated in a research recently performed in Israel. In this country, despite widespread use of vaccination (VC > 93%) and a significant decrease of notifications with aging, serological surveys have shown a considerable circulation of the pathogen in adolescents and elderly [42]. Indeed, serological studies allow one to understand the infective dynamics of *B. pertussis* independently of passive surveillance system and diagnostic bias.

To be effective, surveillance of pertussis should include: passive and active systems; lab tools; coverage data; research on the mixing pattern of the population; *ad hoc* studies on adolescents and adults/elderly; molecular biology epidemiology; mathematical models. At present, we can evaluate, although in an inaccurate way, the epidemiology of pertussis in children, but limited information is available on the incidence, morbidity and risk factors for pertussis in adults, and particularly in elderly. Notwithstanding, a recent study [43] has shown that the incidence of a pertussis notification did not differ by age but hospitalization rates progressively increased. In addition, a review published by Ridda I *et al.* [44] has highlighted that there is a common consensus that pertussis outbreaks are significantly under-recognised in aged care facilities, which are settings where active surveillance data is needed. On the contrary current knowledge of burden of disease is largely based on passive surveillance data. 

Taking into account all these observations, we can conclude that:

1. According to seroepidemiological studies, pertussis is a widespread, even if not adequately measurable, infectious disease.
2. Pertussis continues to be a public-health issue even in countries with high immunization coverage.
3. Immune protection, both natural and vaccine-induced, is not long lasting.
4. Adolescents and adults may become a significant source of infection for unvaccinated or partially immunized newborns.
5. The clinical features of pertussis in adolescents and adults make difficult the diagnosis, which should be based also on lab assessment.
6. Passive surveillance, based on notification, is not a reliable system for the evaluation of pertussis’ incidence and an adequate surveillance should be adopted to evaluate both the burden of infection and the impact of immunization.
7. The paradigm to evaluate before and after the preventive intervention is hardly applicable to pertussis.

The unavailability of comprehensive data should not hamper the adoption of active prophylactic interventions in order to avoid the impact of waning immunity against pertussis [45]; noteworthy, combined vaccines with reduced antigen content (against pertussis as well as tetanus and diphtheria) are suitable for adolescents and adults [11,46].

To avoid an increase of the mean age of acquisition of the infection, a booster dose of low-antigen content combined vaccine should be adopted in adolescents and adults. Some Authors support the use of a decennial booster [13,47,48,49,50].

A decreased risk of infection in newborns can be achieved with the immunization of all family members who could have a strict contact with a newborn (cocoon strategy). This strategy provides a booster for both family members, in the months preceding the birth, and for the mother, immediately after the delivery, and might be combined with a booster targeting adults at high risk of transmitting *Bordetella pertussis* infection to vulnerable infants (e.g., childcare workers, healthcare workers, teachers, ect.). However, although this strategy has been supported by several studies, [51,52,53,54,55], an analysis conducted by Skowronski *et al.* [56], has shown that when pertussis incidence is low, the parental cocoon strategy is resource intensive to prevent serious pertussis outcomes in early infancy, and that the number needed to vaccinate based on local epidemiology should be considered. Finally, Cherry [13] argues that immunizing pregnant women is sound because it reduces the risk that the mother will acquire pertussis and it gives the infant some protection, but women who have multiple pregnancies within a few years may present increased local reactions since vaccine contains tetanus toxoid (*i.e.*, Tdap). He suggests as alternative approach to start DTaP immunization at birth and to complete the first three doses by 3 months of age. In conclusion, the debate is still open and an enhanced surveillance and further studies are needed to fine-tuning pertussis prevention strategy.
